# Intraarticular hip corticosteroid injections offer no meaningful benefit in delaying time to total hip arthroplasty in patients with hip osteoarthritis

**DOI:** 10.1186/s13018-024-05115-x

**Published:** 2024-10-22

**Authors:** Ramesh B. Ghanta, Ellen Tsay, Musa Zaid, Derek Ward, Jeffrey Barry

**Affiliations:** Department of Orthopaedic Surgery, University of California San Francisco, San Francisco, CA USA

**Keywords:** Hip injection, Corticosteroid, Nonoperative treatment, Hip osteoarthritis, Image guided injection, Steroids

## Abstract

**Introduction:**

Symptomatic hip osteoarthritis (OA) causes significant morbidity and functional limitations. While corticosteroid injections (CSI) are commonly offered and administered for OA pain relief, it is unknown if they offer any clinically meaningful long-term benefit or reduce the overall need for surgical intervention.

**Methods:**

A cross-sectional retrospective cohort study was performed on primary hip osteoarthritis patients from a single academic tertiary-care center arthroplasty clinic from 2014 to 2019. Patients were divided into three groups. CSI + THA: hip CSI patients who underwent subsequent ipsilateral THA. CSI-noTHA: hip CSI who have not had ipsilateral THA to date. THA-noCSI: a control group of consecutive hip OA patients who underwent primary THA without prior CSI. Demographic variables, injection relief duration, and radiographic arthritis severity were recorded. Time from clinic presentation to injection and/or THA were compared.

**Results:**

357 patients met inclusion criteria and underwent guided, arthroplasty provider-ordered CSI. Mean duration of relief was 6.7 weeks (SD 8.7). 244 injection patients (67.2%) subsequently underwent THA (CSI + THA). 150 of 390 patients have not undergone THA at mean of 25.5 months follow-up. Mean time from clinic presentation to THA was 8.6 months longer after CSI (16.3, SD 17.8) months in CSI patients compared to 7.7 (SD 10.6) months for patients without CSI (*p* < 0.001). Of 117 patients in the CSI-noTHA group at mean 25 months follow-up, only 43 (12% of all injection patients) had not had THA because they found injections effective. The remaining 74 (63%) of CSI-noTHA patients have been deemed medically unfit for surgery or are currently scheduled for THA.

**Discussion/Conclusion:**

The results of this study suggest the utilization of intra-articular CSI as conservative treatment in an arthroplasty clinic does not prolong time to THA for a clinically important duration. The use of CSI should be reserved for diagnostic purposes and/or short-term pain relief in poor surgical candidates.

**Level of evidence:**

III.

## Introduction

Symptomatic hip osteoarthritis (OA) causes significant morbidity and functional limitations for those who suffer from the disease. An estimated 370,770 primary total hip arthroplasties (THA) were performed in the United States (U.S.) in 2014. By 2030, that number is expected to increase to 635,000 primary hip replacements per year in the U.S [1]. Total hip arthroplasty is the gold standard treatment for end stage hip OA, and is arguably the most successful surgical intervention in all of medicine [2]. Cost-effectiveness and societal benefits from THA have been demonstrated time and again [3–6]. A recent systemic review of the available literature suggests that THAs that are not delayed are more cost-effective than when surgery is delayed [6]. However, even the most successful, predictable surgery comes with inherent risk. As such, patients and providers appropriately seek and exhaust alternative, less invasive treatment options for pain relief prior to committing to surgery.

Oral non-steroidal anti-inflammatory (NSAID) medications and intraarticular corticosteroid injections (CSI) modulate the underlying inflammatory response in OA, and are common options for pain relief [7]. According to the American Academy of Orthopaedic Surgeons Clinical Practice Guidelines, strong evidence supports the use of NSAIDs and CSI to improve short-term pain and/or function in patients with symptomatic osteoarthritis of the hip [8]. While there is ample literature on knee CSI and their efficacy, there is a paucity of data on hip corticosteroid injections likely attributable to the more difficult accurate administration [9, 10]. While these injections are commonly offered and administered for hip OA pain relief, it is unknown if they offer any clinically meaningful long-term benefit or reduce the overall need for surgical intervention. Numerous studies have attempted to examine the efficacy of these injections, but the results have been mixed. Some have demonstrated a significant and prolonged pain relief, while others have demonstrated no relief of symptoms following injection [7, 11, 12]. Additionally, recent studies have called into question the safety of CSI in the hip. Published case reports describe rapid progression of OA following hip injection [13, 14]. Not only are surgeons worried about rapid progression of pre-existing arthritis, but concern exists of increasing the risk of a prosthetic joint infection in THA following a hip CSI [15]. 

The purpose of this study was to determine the effectiveness of hip corticosteroid injections for hip osteoarthritis and if they confer meaningful benefit in extending the time to total hip arthroplasty.

## Methods

Following institutional review board approval, a cross-sectional retrospective cohort study was performed on primary hip osteoarthritis patients from a single academic tertiary-care center arthroplasty clinic from 2014 to 2019. All patients who had undergone image-guided intra-articular hip corticosteroid injections ordered by the orthopaedic providers in the group were identified. An additional control cohort of 100 consecutive THA patients who had not undergone any hip CSI was selected for comparison. Non-operative patients without injections were not included given the heterogeneous nature of presenting diagnoses and states of treatment. For analysis, patients were divided into one of three groups. CSI + THA: hip CSI patients who underwent subsequent ipsilateral THA. CSI-noTHA: hip CSI who have not had ipsilateral THA to date. THA-noCSI: a control group of consecutive primary hip OA patients who underwent primary unilateral THA without history of prior CSI.

All injections were ordered after evaluation by an orthopaedic provider at our institution and performed under fluoroscopic or ultrasound guidance by a musculoskeletal radiologist. Corticosteroid injections were composed of either 40–80 mg methylprednisolone (Depo-Medrol, Pfizer, inc., New York USA) or 40 mg triamcinolone (Kenalong, Bristol-Myers Squibb, Spain, EU) diluted in 1% lidocaine or 0.5% ropivacaine. Patients were excluded for non-primary OA diagnosis, injection ordered by non-orthopaedic providers, injections prior to referral to our clinic, injections performed outside our institution, and/or injections without ultrasound or fluoroscopy guidance. Patient were only included if they had documented follow-up a minimum of 3 months post-CSI.

For each group, the severity of osteoarthritis prior to injection and/or surgery was assessed using the Kellgren-Lawrence (KL) scoring system with patients stratified as early osteoarthritis (KL 1–2) or advanced osteoarthritis (KL 3–4) for sub-analysis [16]. Time from first presentation to the arthroplasty clinic to injection and/or total hip arthroplasty was assessed. It is our practices’ routine to document duration of pain relief on follow-up after injection and this was assessed as well. For patients who underwent an injection without subsequent surgery, the chart was reviewed to determine if there was any reason why the patient ultimately did not undergo surgery (sustained pain relief, poor surgical candidate, currently scheduled awaiting surgery, etc.).

Standard descriptive statistics are reported with a *p*-value of less than or equal 0.05 as cutoff for statistical significance. For bivariate analyses, chi-square or Fisher’s Exact tests were used for categorical data to determine statistical differences. For normally distributed interval or continuous variables a student T-test was used. For non-normally distributed data a Wilcoxon rank sum test was used. There were no external funding sources for this study.

## Results

A total of 390 patients met inclusion criteria and underwent radiographic guided intraarticular hip corticosteroid injection for primary osteoarthritis ordered by our arthroplasty clinic providers. Thirty three patients did not have subsequent follow-up after injection and were excluded leaving 357 patients for analysis. Two hundred and forty of 357 injection patients subsequently underwent ipsilateral THA (CSI + THA), while 117 patients had an injection without subsequent THA at time of study assessment (CSI-noTHA). The control group consisted of 100 consecutive primary total hip arthroplasty patients with no documented injection history (THA-noCSI). On average, patients who underwent corticosteroid injection (with and without total hip arthroplasty) were older than controls that had undergone surgery (Table 1). Additionally, THA patients without injection history (THA-noCSI) had worse radiographic osteoarthritis compared to CSI + THA and CSI-noTHA groups (Table 1). Of patients who had undergone CSI, the average reported duration of pain relief was 6.7 weeks (SD 8.7). OA severity did not correlate with CSI relief duration (KL 1–2 mean 6.7 weeks vs. KL 3–4 6.0 weeks, *p* = 0.69) (Table 2). 32% of CSI + THA and 43% of all CSI patients without THA underwent multiple injections during the study period (mean 2.4 and 2.9 injections, respectively).

**Table 1:** Patient demographics

	Group 1	Group 2	Group 3	*P*-value
**Number of Patients**	240	117	100	
**Average Age**,** Years (SD)**	69.2 (10.5)	72.1 (11.8)	64.5 (13.6)	< 0.005
**Sex**	139 females 100 males	99 females 51 males	42 females 58 males	0.008
**Average Follow-up (months)**	41.4	25.4	26.2	
**Radiographic OA Severity at Presentation**				
**Mild-Moderate OA** ^**1**^ **(KL** ^**2**^ **1–2) (%)**	47 (20)	67 (45)	10 (10)	
**Advanced OA** ^**1**^ **(KL** ^**2**^ **3–4) ** *n* ** (%)**	193 (80)	83 (55)	90 (90)	< 0.005

^1^Osteoarthritis

^2^Kellgren-Lawrence

Group 1 - Patients with corticosteroid injection then subsequent total hip arthroplasty

Group 2 - Patients with corticosteroid injection and no subsequent total hip arthroplasty

Group 3 – Control group of patients who underwent total hip arthroplasty without injection

**Table 2:** Average duration of pain relief (weeks) following intraarticular hip injection and frequency of multiple injections

	Group 1	Group 2	*P*-value
**Mean duration pain relief**,** weeks (SD)**	6.7 (8.2)	6.0 (7.9)	0.69
**Patients who underwent multiple injections**,* n*** (%)**	79 (32)	41 (27)	0.08
**Average number of additional injections**,* n*	2.4	2.85	

Group 1 - Patients with corticosteroid injection then subsequent total hip arthroplasty

Group 2 - Patients with corticosteroid injection and no subsequent total hip arthroplasty

Group 3 – Control group of patients who underwent total hip arthroplasty without injection

The mean time from initial arthroplasty clinic presentation to THA was 16.3 (SD 17.8) months in patients who had gotten CSI compared to 7.7 (SD 10.6) months for patients without CSI, *p* < 0.001. When broken down by arthritis severity, there was a statistically significant difference in time to THA for advanced OA patients after CSI (14.9 (SD 16.9) months vs. 7.5 (SD 7.2) months, *p* < 0.001). There was no significant difference, however, when time from presentation to first injection was removed from the calculation (CSI + THA injection to THA mean 10.1 months vs. THA-noCSI presentation to THA mean 7.6 months, *p* = 0.14). Among CSI + THA patients, 77% had the surgery within one year of their first injection.

For the CSI-noTHA patients, mean follow-up was 19.1 months (SD 17.7 months). Forty eight of 117 patients (41%) were deemed to be medically not surgical candidates, and 26/117 patients (22%) are currently scheduled and awaiting THA, leaving just 43/117 patients (37%) who have found non-operative treatment sufficient (Table 3). When looking at all 357 patients who had undergone hip corticosteroid injections, only 43/357 (12%) had not had a THA to date because they found the injections effective.

**Table 3:** Breakdown of patients who underwent an isolated hip corticosteroid injection and no subsequent arthroplasty

	Group 2 (all)	Group 2 (mild-moderate OA)	Group 2 (severe OA)
**Number of patients**	150	68	82
**Found CSI helpful and non-operative treatment sufficient**,* n*** (%)**	43 (29)	25 (37)	18 (22)
**Medically not a surgical candidate**^**1**^, *n*** (%)**	48 (32)	17 (25)	31 (38)
**Currently scheduled and awaiting surgery or surgery confirmed at OSH**,* n*** (%)**	26 (17)	5 (10)	21 (26)
**No post injection follow up in arthroplasty clinic**,* n*** (%)**	33 (22)	20 (29)	13 (16)

Group 2 - Patients with corticosteroid injection and no subsequent total hip arthroplasty

^1^not surgical candidate for variety of reasons, including elevated BMI or decompensation of medical co-morbiditis (cancer, cardiopulmonary disease)

**Fig. 1:**
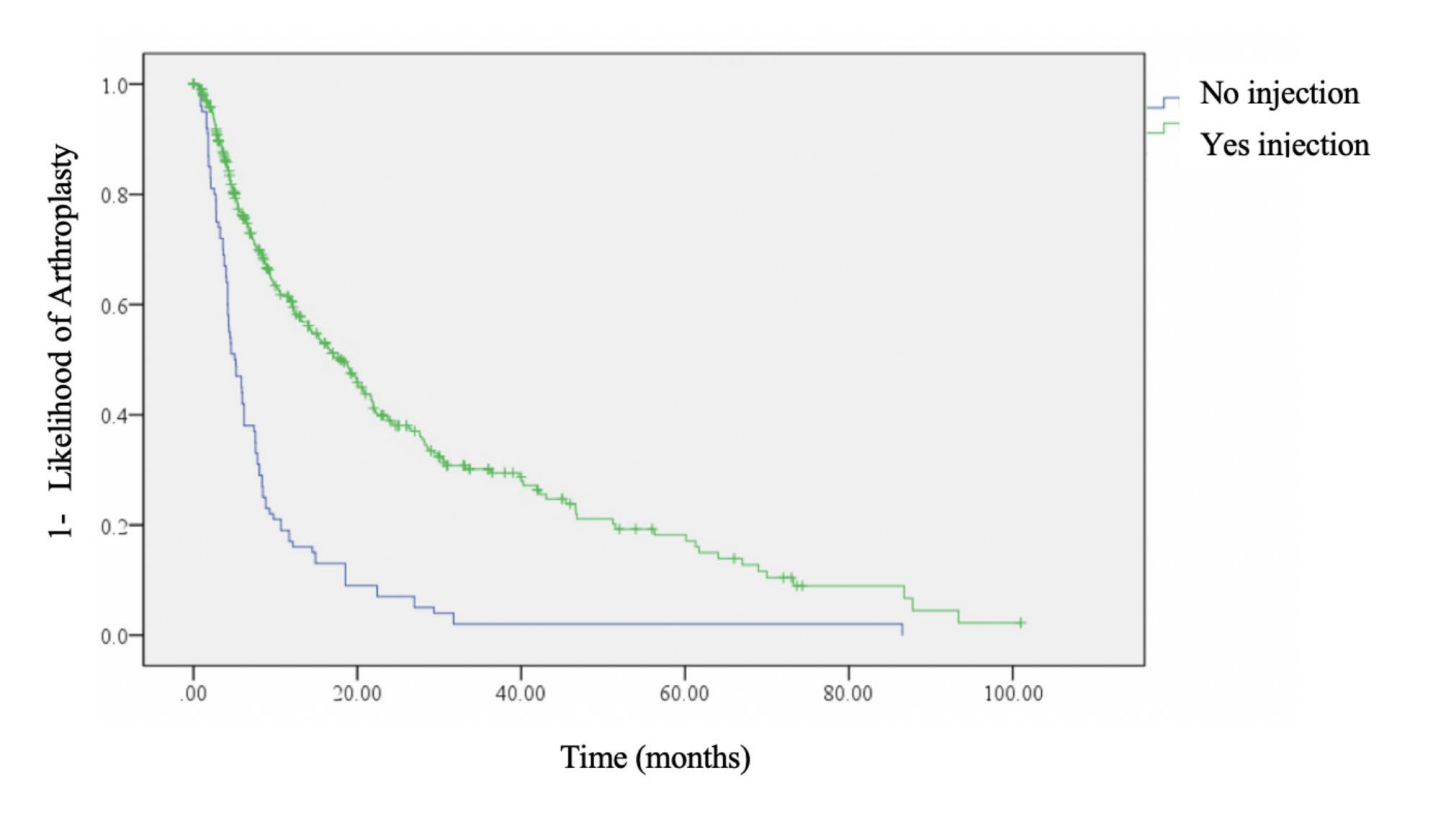
Kaplan-Meier survival analysis for time to surgery (TTS) from initial presentation to arthroplasty clinic for patients who underwent injection (green) and those who had no injection (blue) (*p* < 0.0005)

Figure 1 demonstrates survivorship from clinic presentation to THA comparing patients with and without CSI. For this Kaplan Meier analysis patients deemed medically unable to undergo surgery were excluded from the CSI group numbers.

## Discussion

Hip osteoarthritis causes significant morbidity and loss of function for millions each year. While total hip arthroplasty offers excellent pain relief and good return to function, many physicians work with patients to optimize non-operative treatments prior to proceeding with THA. The results of this study suggest that the use of an intraarticular corticosteroid injection by orthopaedic arthroplasty surgeons does not prolong the time to THA for a clinically important duration for most patients with only 12% of injection patients finding them useful enough to avoid surgery at an average of 25 months follow-up.

The American Academy of Orthopedic Surgeon’s clinical practice guidelines currently support the use of intraarticular steroid injections for the short-term treatment of hip osteoarthritis with a strong recommendation based off of three randomized controlled trials which showed short term improvement in pain when compared with saline placebo [8]. While some studies have demonstrated up to three months of pain relief, other have only demonstrated less than 1 week of pain relief following an injection [12, 17]. In our study, we found that the average duration of pain relief was 6 weeks and over 75% of the study population underwent THA within 1 year of their injection. Given the short duration of relief and high conversion to THA within a year we do not consider this an effective treatment modality for the hip in most patients with respect to the eventual need for a THA. There is certainly an argument to be made that an increase in time to THA by up to 9 months can have positive value in patients psychologically coming to terms with the need for hip replacement and feeling more comfortable knowing they have exhausted all conservative options prior to surgical management. However, with an average of 6 weeks of pain relief per injection, it is possible that this delay in time to THA is spent in significant pain that could otherwise have been addressed by a total hip replacement.

Comparing the literature surrounding the hip to a joint with similar treatment paradigms, injections for knee osteoarthritis may also not provide the benefit previously thought. While considered generally safe, intra-articular corticosteroid injections for symptomatic knee osteoarthritis may also not provide a meaningful benefit in reducing pain and may actually accelerate cartilage loss. A recent randomized controlled trial comparing intra-articular saline to corticosteroid found no difference in pain control after 2 years and increased cartilage loss when measured with MRI [18]. Despite this new data, a Cochrane Review from 2015 did show improvements in pain and function following intra-articular knee corticosteroid injection [19]. 

Injections also come with cost and risk. As opposed to knee injection, hip injection requires imaging guidance for accurate administration, thereby increasing both systemic costs and patient costs related to additional appointments, travel, and post-procedural care. Recent case series have also brought to light the previously unrecognized phenomenon of rapid progression of OA following injection [13, 14]. Additionally, it has been demonstrated that these injections place patients at an increased risk of developing a prosthetic joint infection in the future [15]. This infection risk decreases over time. However, with such short clinical benefit on average, injections may delay surgical treatment unnecessarily. This study was not powered to investigate or examine the rates of postoperative complications, such as infection following injection or the development of rapidly progressive osteoarthritis, however, the current protocol at our institution is to wait 3 months following an injection prior to replacing that joint. The average time to surgery may also be artificially inflated due to this protocol.

There are still clinical scenarios and patient populations in whom intraarticular hip CSI will remain an important diagnostic and therapeutic tool. The authors still find intraarticular injections beneficial, especially in cases of diagnostic ambiguity, such as patients with overlapping spinal pathology [20]. In such patients, delineating the true location and contribution percentage of their pain from the hip, especially during the anesthetic portion of an intraarticular injection can be invaluable. Furthermore, a large number of patients in our study who underwent an injection without having a subsequent THA have been deemed to be poor surgical candidates due to medical comorbidities. While the duration of pain relief may be limited, this population may be appropriate for this therapy, as it is lower risk than THA and offers at least some, albeit limited, symptomatic relief. The findings of this study may be most useful in counseling patients who are considering an injection in hopes of delaying a total hip arthroplasty. A minority of injection patients (12%) found the injections helpful enough to meaningfully delay THA at an average 2 years follow-up.

This study is not without limitations. First and foremost, this study only examined patients who were referred for injection from the arthroplasty clinic. We excluded patients who were referred for injection from their primary care physicians, as the appropriateness, quality, duration of relief and technique of the injections could not be assessed. This may represent a population of patients with less severe osteoarthritis that could benefit from injections. The retrospective nature of the study also introduces significant selection bias. Additionally, the no injection group in our survival analysis only included eventual surgical patients and not all patients evaluated in our arthroplasty clinic. Furthermore, in comparisons of patient with and without CSI, we have not included patients with hip osteoarthritis who did not undergo injection and have not undergone THA, as we felt that group would introduce such heterogeneity as to make interpretation and applicability to clinical practice difficult.

## Conclusions

In conclusion, intraarticular hip CSI injection does not provide long term pain relief or meaningfully prolong the time to THA in most patients with primary hip osteoarthritis. The use of this non-operative treatment modality should be reconsidered and reserved only for poor surgical candidates or when needed for diagnostic purposes.

## Data Availability

No datasets were generated or analysed during the current study.
